# 3D-Printed Mucoadhesive Collagen Scaffolds as a Local Tetrahydrocurcumin Delivery System

**DOI:** 10.3390/pharmaceutics13101697

**Published:** 2021-10-15

**Authors:** Mireia Andonegi, Teresa Carranza, Alaitz Etxabide, Koro de la Caba, Pedro Guerrero

**Affiliations:** 1 BIOMAT Research Group, Escuela de Ingeniería de Gipuzkoa, University of the Basque Country (UPV/EHU), Plaza de Europa 1, 20018 Donostia-San Sebastián, Spain; mireia.andonegui@ehu.eus (M.A.); alaitz.etxabide@ehu.eus (A.E.); 2 Domotek SL, B° Santa Luzi 17, 20400 Tolosa, Spain; teresa@domotek.es; 3 BCMaterials, Basque Center for Materials, Applications and Nanostructures, UPV/EHU Science Park, 48940 Leioa, Spain; 4 Proteinmat Materials SL, Avenida de Tolosa 72, 20018 Donostia-San Sebastian, Spain

**Keywords:** native collagen, 3D printing, sustained release

## Abstract

Native collagen doughs were processed using a syringe-based extrusion 3D printer to obtain collagen scaffolds. Before processing, the rheological properties of the doughs were analyzed to determine the optimal 3D printing conditions. Samples showed a high shear-thinning behavior, reported beneficial in the 3D printing process. In addition, tetrahydrocurcumin (THC) was incorporated into the dough formulation and its effect on collagen structure, as well as the resulting scaffold’s suitability for wound healing applications, were assessed. The denaturation peak observed by differential scanning calorimetry (DSC), along with the images of the scaffolds’ surfaces assessed using scanning electron microscopy (SEM), showed that the fibrillar structure of collagen was maintained. These outcomes were correlated with X-ray diffraction (XRD) results, which showed an increase of the lateral packaging of collagen chains was observed in the samples with a THC content up to 4%, while a higher content of THC considerably decreased the structural order of collagen. Furthermore, physical interactions between collagen and THC molecules were observed using Fourier transform infrared (FTIR) spectroscopy. Additionally, all samples showed swelling and a controlled release of THC. These results along with the mucoadhesive properties of collagen suggested the potential of these THC–collagen scaffolds as sustained THC delivery systems.

## 1. Introduction

In recent decades, biofabrication of tissue constructs with hierarchical architecture has acquired special interest in tissue engineering and regenerative medicine as biological substitutes that can assist and promote the tissue healing process, providing the optimum conditions for the re-establishment of the damaged tissue [1]. In that way, different biofabrication methods have been developed, such as freeze-drying, electrospinning, and microengineering. However, due to the complex geometry of native tissues, these methods have shown limited reproducibility and versatility in the fabrication procedures [2]. More recently, 3D printing has emerged as a novel biofabrication method, characterized by a computer-based control that provides the addition of material, layer by layer, to obtain constructs with specific shapes [3]. 3D printing offers automated material deposition with spatial control and high reproducibility, which allows for the recreation of preprogrammed 3D tissue constructs, with personalized characteristics that mimic the patient-specific architecture and, consequently, the function of targeting tissues [4]. In addition, the technology and the low temperature used in 3D printing provide the potential to create scaffolds based on biopolymeric hydrogels with controlled placement of living cells [5], drugs [6], and/or bioactive molecules [7].

Collagen hydrogels have numerous attractive features for use as tissue engineering scaffold materials. Collagen is the main structural protein in the extracellular matrix (ECM) of mammalians and is a major determinant of the architecture and strength of many tissues [8]. Besides, collagen participates in numerous physiological interactions through its specific cell-binding sites, where the cell attachment, proliferation, migration, growth, and differentiation are the most significant [9,10]. Thus, collagen scaffolds have been used for tissue engineering due to their hemostatic, low antigenic, biodegradable, and biocompatible properties [11,12]. However, the resulting scaffolds offer poor mechanical properties and structural stability [13]. Hence, different strategies have been used to overcome these limitations on the overall integrity of printed collagen, incorporating additives that induce physical or chemical crosslinking [14].

Concerning additive incorporation, thetrahydrocurcumin (THC) can be incorporated into printable collagen formulations to obtain active hydrogels for wound healing. THC is a plant-derived compound obtained from the hydrogenation of curcumin that has attracted special interest in the pharmaceutical and cosmetic industries [15,16]. Due to the poor solubility in water, low absorption from the gut, rapid metabolism, and rapid systemic elimination of curcumin, its health benefits are limited [17]. Likewise, several studies have analyzed the antibacterial [18], antidiabetic [19], anticancer [20], and anti-inflammatory activities [21] of THC metabolite using several drug-delivery systems, such as polymeric nanoparticles or capsules, solid lipid nanoparticles, liposomes, microemulsions, nanovesicles, and foams [22,23]. Furthermore, THC can be beneficial for local treatments, since it enhances collagen deposition, ECM accumulation, fibroblast growth, vascular density, and angiogenesis, contributing to faster wound healing [24].

Collagen hydrogels are printable if low concentrations, usually lower than 10 mg/mL [25], are used. In contrast to the usual use of these low collagen concentrations (≈2 mg/mL), in this study THC–collagen scaffolds were obtained by 3D printing using concentrated collagen solutions (200 mg/mL). Therefore, the optimization of the 3D-printing process, based on rheological analysis, had to be performed. It is worth noting that the use of crosslinkers was not needed since native collagen with no chemical pretreatment was used and, thus, the collagen triple helix structure was maintained. On the other hand, to analyze the effect of different THC concentrations on the scaffolds, physicochemical, thermal, morphological, and barrier properties of 3D-printed scaffolds were assessed. With the aim of a potential use of these scaffolds for wound healing applications, the mucoadhesive properties of the scaffolds were also evaluated.

## 2. Materials and Methods

### 2.1. Materials 

Porcine collagen was supplied by Tenerias Omega (Navarre, Spain) and tetrahydrocurcumin (THC) was gifted by Sabinsa Corporation (East Windsor, NJ, USA).

### 2.2. Mixture Preparation 

Native collagen was obtained according to the method of Andonegi et al. [26]. Firstly, porcine skins were defatted by immersion into 1 M NaOH solution for 12 h and then, neutralized in phosphate buffer saline (PBS) solution (pH = 7.4). Afterwards, collagen was ground and freeze-dried to facilitate the subsequent processing. After this treatment, 2.5 g of collagen, THC (0, 2, 4, 6 wt% based on dry collagen) and 0.5 M acetic acid (1:5 collagen/acetic acid ratio) were mixed using an ultra-turrax T25 (IKA, Staufen, Germany) until homogeneous pastes were achieved. The mixing process (2000 rpm, 2 min) was carried out in a cold bath to prevent the dough from heating up. Finally, the mixtures were stored in light-protected syringes at 4 ± 1 °C until use. Four systems were produced and designated as Control, THC2, THC4, and THC6, as a function of THC content, 0, 2, 4, and 6 wt%, respectively.

### 2.3. Rheological Evaluation 

The viscoelastic properties of THC–collagen mixtures were studied at 35 °C by a Thermo Scientific Haake Rheostress1 Rheometer (IFI S.L., Vigo, Spain), equipped with a serrated plate–plate geometry (diameter of 35 mm). The gap between plates was 1 mm. Firstly, strain sweep tests were carried out at a constant frequency of 1 Hz and between 0.01% and 100% strain to determine the linear viscoelastic range (LVR) and the critical strain of the LVR. Subsequently, the frequency sweep test at a strain within the LVR range was carried out between 0.01 and 50 Hz to obtain loss tangent (tan δ), elastic (G’), and viscous (G”) moduli. Finally, the shear flow test was carried out in the shear rate (γ ˙) range from 0.1–50 s^−1^ using the same probe and gap. Samples were left running for 5 min before the test started to stabilize temperature and allow residual stress to relax.

The obtained flow data were fitted to the Williamson model for shear-thinning materials:

(1) $$\eta =\frac{\eta_{0}}{1+(k\cdot {\dot{\gamma )}}^{\left(1-n\right)}}$$

where η is the viscosity, η_0_ is the limiting viscosity at low shear rate, k is the consistency coefficient, and n is a flow index [27].

Additionally, a master curve of flow rate was obtained in order to describe the flow behavior of the doughs. A time-concentration superposition was made; first, using a vertical shift, given by normalization with the η_0_ value of each dough; then, a horizontal shift specified by a time-concentration factor, a_c_ [28]. Finally, the master curve was fitted to Williamson’s model:

(2) $$\eta =\frac{\eta_{0}}{1+(k\cdot a_{c}\cdot {\dot{\gamma )}}^{\left(1-n\right)}}$$

Moreover, the flow behavior of collagen doughs at the 3D-printing conditions used can be assessed by the Weissenberg–Rabinowitsch equation:

(3) $${\;\gamma \;˙}_{w}={\;\gamma \;˙}_{\mathrm{wN}}\frac{\left(3n+1\right)}{4n}$$

where *n* is the flow index, ɣ̇_w_ is the shear rate at the wall, ɣ̇_wN_ = (8V/D) is the nominal shear rate, V is the average inlet velocity of the fluid, and D is the nozzle diameter [29].

### 2.4. 3D Printing 

Cura (Ultimaker Cura 4.6.1 Software, Utrecht, The Netherlands) was used to design the scaffolds as cylindrical mesh (21 mm diameter; 0.9 mm height) with infill of 75% (340 μm pore diameter). Collagen scaffolds were fabricated at a printing temperature of 35 °C using a syringe-based extrusion 3D DomoBIO printer (Domotek, Tolosa, Spain). The 3D printing speed was set at 3 mm/s through a G18 nozzle with an inner diameter of 0.84 mm. The temperature of the 3D-printer bed was fixed at 25 °C with a layer height of 0.3 mm. No post-processing treatment was carried out. All samples were left to dry and conditioned in a climatic chamber at 25 °C and 50% relative humidity before testing.

### 2.5. Fourier Transform Infrared (FTIR) Spectroscopy 

Fourier transform infrared (FTIR) spectra were performed by using a Nicolet 380 FTIR spectrometer (Nicolet Instrument, Barcelona, Spain) equipped with attenuated total reflectance (ATR) crystal (ZnSe). A total of 32 scans were carried out at 4 cm^−1^ resolution.

### 2.6. Differential Scanning Calorimetry (DSC) 

DSC measurements were performed by a Mettler Toledo DSC 822 (Mettler Toledo, Barcelona, Spain). Samples (3 mg) were put into sealed aluminium pans, to prevent mass loss, and subjected to a heating ramp from 25 °C to 250 °C at a rate of 10 °C/min, under nitrogen atmosphere to avoid oxidation.

### 2.7. X-ray Diffraction (XRD) 

XRD measurements were performed by a diffraction unit PANalytical Xpert PRO (PANalytical BV, Almelo, The Netherlands), operating at 40 kV and 40 mA. The radiation was generated from a Cu-Kα (λ = 1.5418 Å) source. The diffraction data were collected from 2θ values from 2° to 50°, where θ is the angle of incidence of the X-ray beam on the sample.

### 2.8. Scanning Electron Microscopy (SEM) 

Scaffolds were placed on a metal stub and coated with gold using a JEOL fine-coat ion sputter JFC-1100 (JEOL Ltd., Tokyo, Japan), under argon atmosphere. Samples were observed using a Hitachi S-4800 scanning electron microscope (Hitachi, Madrid, Spain) at 15 kV accelerating voltage.

### 2.9. Ultraviolet-Visible (UV–Vis) Spectroscopy and THC Release 

The light-barrier properties were determined using a UV–Vis Multiskan SkyHigh (Thermo Fisher, Madrid, Spain) spectrophotometer to measure the light absorption at wavelengths ranging from 200 to 800 nm.

UV–Vis spectroscopy was used to determine the THC release in PBS solution (pH = 7.4). Firstly, the wavelength of maximum absorbance for THC in PBS was measured (λ_max_ = 280 nm) and then, standard THC solutions were prepared over a concentration range of 3.906–1000 ppm to establish a calibration curve (y = 0.0004x + 0.0683; R^2^ = 0.9635).

THC release was determined by immersing the scaffolds into PBS solution (10 mL) at room temperature for 2 d. Aliquots (3 mL) were withdrawn at specific times (4, 6, 8, 24, 30, and 48 h), replaced with fresh buffer and analyzed by UV–Vis spectroscopy at 280 nm. All tests were carried out in triplicate for each composition.

The THC release data were kinetically evaluated by Korsmeyer–Peppas model:

(4) $${\frac{M_{t}}{M}}_{\infty }=\;kt^{n}$$

where M_t_/M_∞_ is the fraction of drug released at time *t* and k is Korsmeyer–Peppas constant related to the properties of the delivery system, such as structural and geometric properties. *n* is the release exponent that shows the THC release mechanism: *n <* 0.45, a pseudo-Fickian diffusion mechanism; *n* = 0.45 a Fickian mechanism; 0.45 < *n* < 0.89, an anomalous diffusion mechanism; and *n =* 0.89, a non-Fickian diffusion mechanism [30].

### 2.10. Water Uptake (WU) 

Scaffolds were weighed (w_i_) and then immersed into PBS (pH = 7.4). Samples were reweighed at specific times (w_t_), 0, 15, 30, 45, 60, 90, 120, 180, 240, 300, 360, 420, 480, 540, 1440, and 1800 min, until constant values were achieved. The water uptake was calculated as:

(5) $$\mathrm{WU}\;\left(\%\right)=\frac{w_{t}{-w}_{i}}{w_{i}}\times 100$$

Measurements were carried out for three specimens of each sample.

### 2.11. In Vitro Mucoadhesion Study 

Mucoadhesive properties of collagen scaffolds were determined using TA.XT.Plus C Texture Analyzer (Aname Instrumentación Científica, Madrid, Spain), equipped with a 5 kg load cell and a 3.5 mm diameter cylinder probe. Type II mucin from porcine stomach (Sigma-Aldrich, Madrid, Spain) was used as a biological substrate. Before testing, a filter paper was hydrated by immersion into a PBS solution of type II mucin (1.0 wt%) for 5 min at 37 °C.

The excess surface liquid was withdrawn and then the substrate was horizontally kept on the cylindrical probe. Samples of each formulation were packed into cylindrical vessels (15 mm diameter) and placed on an upper cylinder probe lowered at a constant speed of 1 mm/s until the mucoadhesive surface was reached. After keeping a contact time of 30 s under a force of 0.2 N, the probe with the attached sample was removed at a constant rate (1 mm/s). Texture Exponent 32 software was used to determine the maximum detachment force (F_max_) and the work of adhesion (W_adh_). All measurements were performed with at least five replicates.

### 2.12. Statistical Analysis 

To determine significant differences between samples, analysis of variance (ANOVA) was done with SPSS software (SPSS Statistic 25, IBM Corp., Armonk, NY, USA). Tukey’s test with a statistically significance at the *p* < 0.05 level was considered for multiple comparisons among different systems.

## 3. Results and Discussion

First of all, rheological behavior was analyzed to optimize the 3D-printing process of collagen scaffolds. Additionally, to study the effect of different THC concentrations, physicochemical, thermal, morphological and barrier properties of 3D-printed scaffolds were assessed. Finally, keeping in mind a suitable application of these scaffolds, their mucoadhesive properties were evaluated with the aim of providing relevant information to highlight the potential use of these scaffolds for wound healing.

### 3.1. Rheological Properties

**Figure 1:**
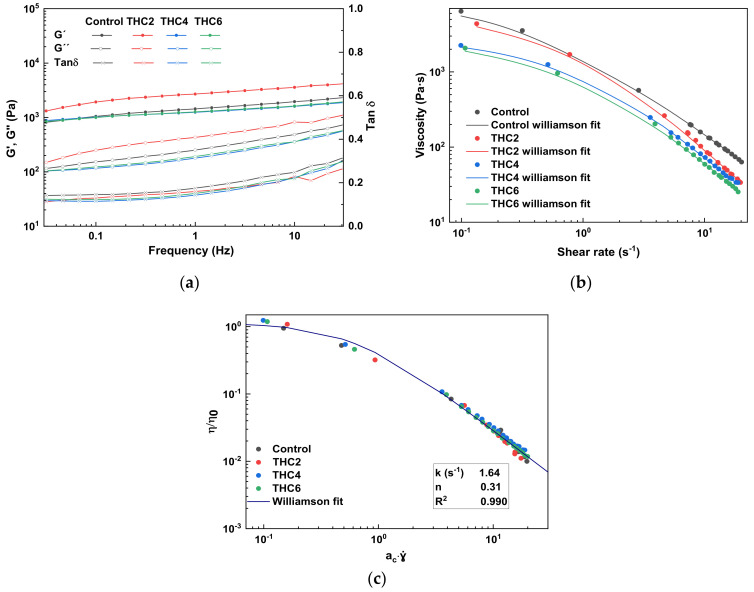
(**a**) Storage moduli (G´), loss moduli (G”), and loss tangent (tan δ), obtained through frequency sweep test for collagen doughs; (**b**) Flow curves of collagen doughs, fitted by Williamson model; and (**c**) Master flow curve for collagen doughs fitted by Williamson model, after performing a THC concentration-dependent shift (η_0_, a_c_) using the control sample as reference.

The knowledge and control of rheological properties are of great relevance to analyze and design the 3D-printing process [31]. Hence, stress sweep, frequency sweep, and flow tests were performed to analyze rheological properties before 3D printing. First, stress sweep tests were performed to determine the linear viscoelastic range (LVR) of THC–collagen doughs and then, frequency sweet tests were carried out within the LVR. The results are shown in Figure 1a. A predominant elastic behavior was observed in all samples since the storage modulus (G’) was greater than the loss modulus (G”) in the studied interval [32]. Additionally, small dependence on frequency was observed in the loss modulus, while the storage modulus remained nearly constant, demonstrating the system stability. It is worth noting that the collagen network structure did not collapse, since no crossover point between G’ and G” was observed. This behavior followed the typical response of protein-based hydrogels observed in several studies [33,34]. Furthermore, the hydrogel behavior did not show noteworthy differences with the addition of THC, since similar tan δ (G”/G´) values were obtained for all the formulations. Likewise, the dough printability can be assessed in terms of the minimum pressure required and modelled using the loss tangent. The loss tangent values found in this study ranged from 0.15 to 0.40, in accordance with other studies that suggested good printability within loss tangent values from 0.25 to 0.45 [14].

The viscosity suitable for 3D printing must be low enough to permit easy extrusion through the nozzle and high enough to be cohesive with the previously deposited layer while maintaining the shape [35]. Therefore, the flow behavior of THC–collagen samples was analyzed and the dependence of apparent viscosities on shear rate is shown in Figure 1b. All samples displayed similar steady-state viscosity (η) pattern of non-Newtonian fluids with a shear-thinning behavior and a tendency to reach a Newtonian region at a low shear rate (Newtonian plateau zone); thus, zero-shear viscosity (η_0_) was estimated [36]. Zero-shear viscosity is the viscosity when the material is at rest; this is a limiting value that cannot be measured directly and must be estimated by extrapolation, fitting the data to the model. The experimental flow data obtained were satisfactorily fitted (R^2^ > 0.996) to the Williamson model (Equation (1) and the resulting fit parameters are shown in Table 1. The shear-thinning performance indicated that collagen chains were affected by the shear stress between the layers when the flow rate increased, thereby reducing the force between them [37]. This behavior was reported beneficial in the 3D-printing process for solid-like materials to be extruded through the nozzle [28,38]. As can be seen in Table 1, all samples showed relatively low flow index values (*n* < 0.42), corroborating the shear-thinning behavior of the samples [39]. Furthermore, the decrease of η_0_ with the increase of THC concentration denoted the establishment of a lower number of links between collagen and THC molecules [40]. 

As all samples showed a similar tendency in the shear-thinning region, curves could be fitted to a single master curve (Figure 1c). The shift factor (a_c_) was calculated and included in Table 1, where a_c_ = 1 was assigned to the control sample. A decrease of a_c_ from 1 to 0.67 in THC4 and THC6 doughs indicated a higher consistency when the THC content increased [28]. Therefore, the relatively small tan δ and the low viscosity observed at high shear rates suggested that the THC–collagen doughs would be easily extruded by the syringe with a high shear rate at the nozzle tip [41].

**Figure 2:**
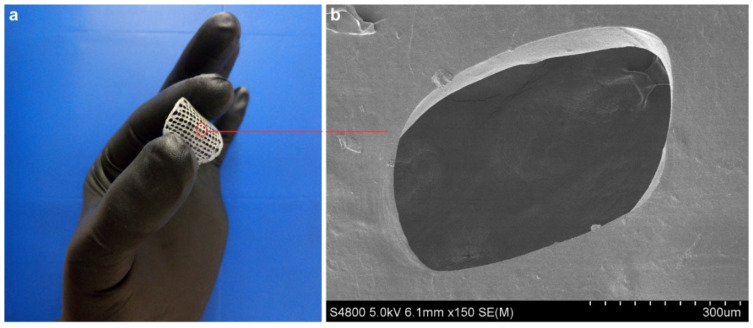
(**a**) A 3D printed THC6 scaffold; and (**b**) SEM image of THC6 surface.

For a further assessment of the flow behavior of collagen doughs in the 3D-printing process, the shear rate in the nozzle tip was calculated (Equation (3) and the values are shown in Table 1. Once the flow curves were obtained and the shear-thinning behaviour observed, high shear rate values were selected since, under these conditions, the material offers less resistance (lower viscosity) and flows better. As the flow index (n) of the master curve was obtained by fitting data to the Williamson model, the printing velocity could be obtained using the equation of Weissenberg-Rabinowitsch (Equation (3). 3D-printed scaffolds are shown in Figure 2.

### 3.2. Physicochemical and Thermal Properties

**Figure 3:**
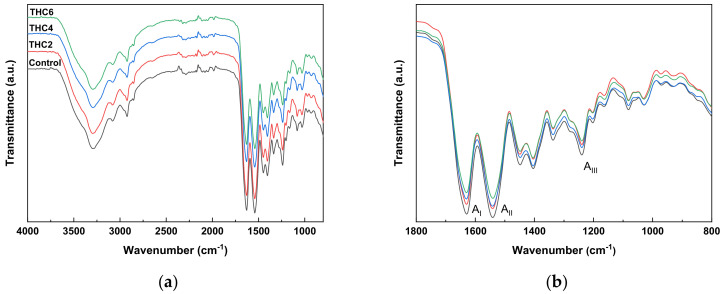
FTIR spectra of 3D-printed collagen scaffolds: (**a**) from 4000 to 750 cm^−1^; and (**b**) from 1800 to 750 cm^−1^.

To assess the interactions among the components of the scaffold formulation, FTIR analysis was carried out and FTIR spectra are shown in Figure 3.

**Figure 4:**
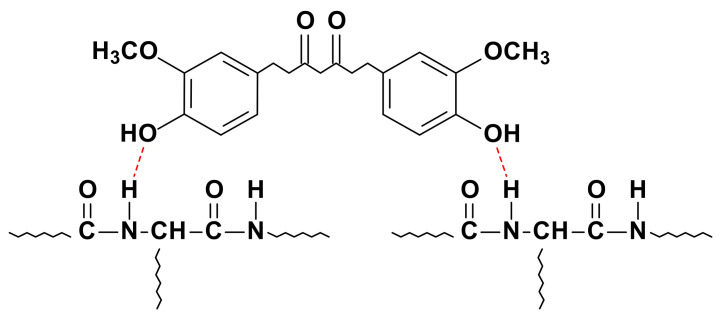
Schematic diagram of the interactions between collagen and THC by hydrogen bonding.

All the spectra showed the main absorption bands assigned to the peptide bonds in collagen: N–H stretching vibration of amide A at 3287 cm^−1^, C=O stretching of amide I at 1630 cm^−1^, N–H bending of amide II at 1540 cm^−1^, and C–N stretching of amide III at 1240 cm^−1^ [42]. The amide A band is commonly observed in the wavenumber range of 3400–3440 cm^−1^ but, when the band position is shifted to a lower frequency, this shift indicates that N–H groups in collagen are involved in hydrogen bonding (Figure 4), leading to stability in the collagen triple-helical structure [43]. Additionally, a change in the intensity of the amide bands was observed with the incorporation of THC, indicative of physical crosslinking. No more obvious changes were observed in FTIR spectra, suggesting the prevalence of collagen secondary structure after the mixture preparation and 3D-printing processes [44].

**Figure 5:**
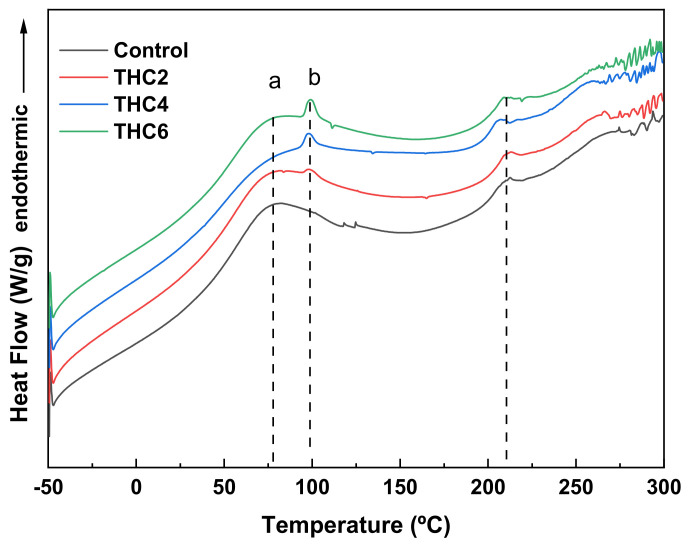
DSC thermograms of collagen scaffolds: (**a**) free and bound water release, (**b**) THC melting, and (**c**) collagen thermal denaturation.

The thermal behavior of collagen scaffolds was studied using DSC analysis. As can be seen in Figure 5, all samples exhibited two endothermic peaks: the first peak, around 82 °C, is related to the free and bound water release [45]; and the second peak, around 210 °C, is associated with collagen thermal denaturation process [46]. During this transition, collagen experienced conformational changes from triple helix to random coil and the structural water was released [47,48,49]. Therefore, it is worth noting that these values confirmed the prevalence of the triple helix structure of collagen after THC addition and 3D printing, as also observed by FTIR analysis. Additionally, samples with THC showed a small peak around 95 °C, related to THC melting [23], and the enthalpy value increased with THC content.

### 3.3. Morphological and Barrier Properties

**Figure 6:**
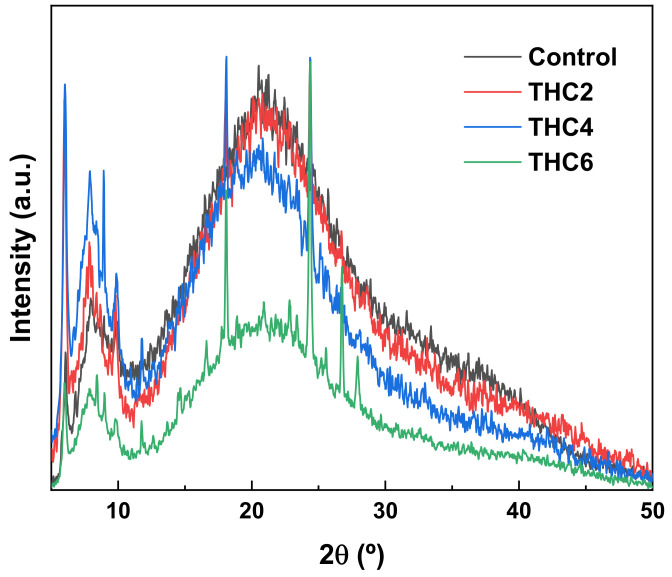
XRD patterns of collagen scaffolds.

For a further analysis of the effect of THC addition on collagen structure, XRD and SEM analyses were carried out. As for XRD analysis (Figure 6), all samples showed XRD patterns of nearly amorphous materials. The peak around 70°, related to the triple helix structure of collagen, represents the lateral packing distance between collagen chains [50] and the broad peak around 20°, associated with the diffuse scattering of collagen fibers, represents the amorphous structure of the samples [51]. Furthermore, the samples with THC showed the characteristic peaks of THC at 18° and 24° [15]. As can be seen, all samples showed similar XRD patterns, indicating the prevalence of the collagen structural order. When a higher THC content was added (THC6), the peak intensity at 7° and 20° decreased, suggesting the decrease of the structural order in collagen scaffolds.

**Figure 7:**
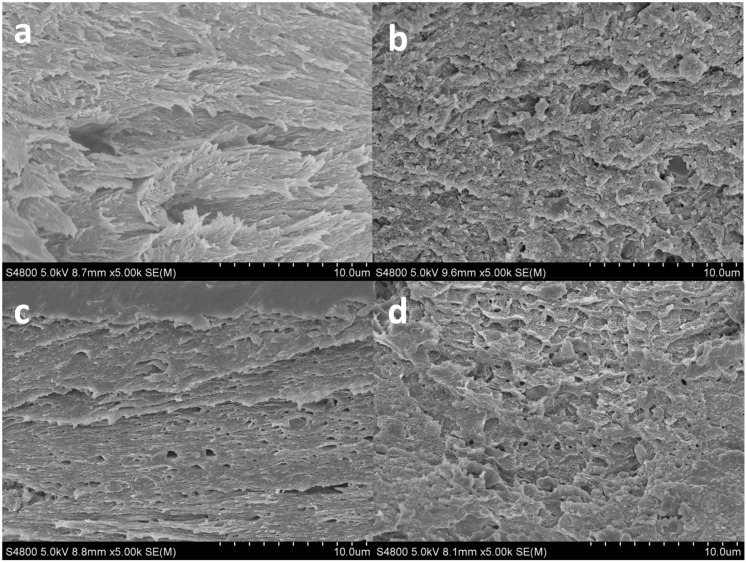
SEM images of the cross-section of collagen scaffolds: (**a**) control; (**b**) THC2; (**c**) THC4; and (**d**) THC6.

To assess the morphology of 3D-printed scaffolds and ensure a good comprehension of the microstructure of the scaffold, SEM analysis was carried out and cross-section images are shown in Figure 7. SEM micrographs showed that all samples had a similar compact amorphous structure. When THC content increased from 4 to 6 wt%, a less organized structure was observed, in accordance with XRD results. It is worth noting that 3D-printed layers cannot be differentiated, indicating their good adhesion.

**Figure 8:**
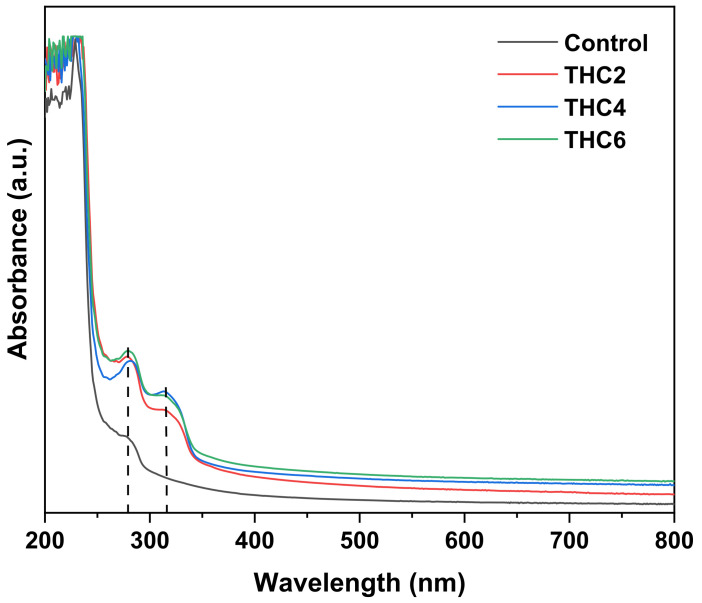
UV–Vis spectra of collagen scaffolds.

Considering wound dressing as a feasible application of these scaffolds, light barrier properties were analyzed by UV–Vis spectroscopy. As can be seen in Figure 8, control samples showed the characteristic UV light barrier of collagen with an absorbance maximum from 200 to 250 nm, associated with carbonyl, carboxyl and amide groups, and a small peak between 250 and 280 nm, related to chromophores groups of tyrosine and phenylalanine amino acids [52,53]. The addition of THC showed a slight increase of the absorbance in the visible light range (400–800 nm) and a strong increase in the UV range (200–400 nm) due to the absorption peak of THC around 280 nm with a shoulder at 310 nm [54]. Furthermore, the increase of the absorbance around 210 nm may be related to the photo-oxidized THC [55]. Nevertheless, no relevant difference was observed among collagen scaffolds with THC.

### 3.4. Water Uptake (WU) and THC Release

**Figure 9:**
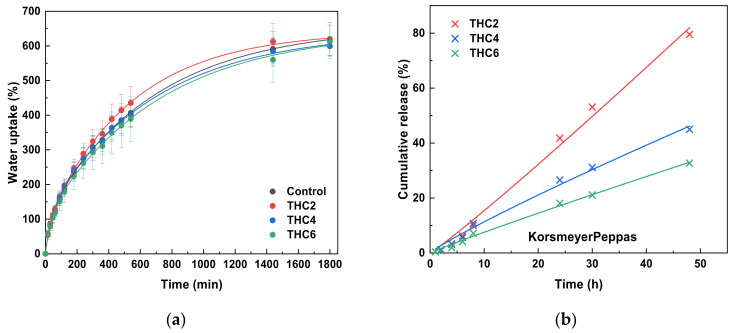
(**a**) Water uptake and (**b**) THC release from THC–collagen scaffolds (data fitted to Korsmeyer–Peppas model).

WU measurements were performed to determine the effect of THC on the water absorption capacity of the scaffolds. WU results showed the capacity of collagen scaffolds to hold a large number of water molecules. As can be observed in Figure 9a, THC concentration had no relevant effect on the WU capacity, which was around 600%. The continuous growth of WU values occurred until 1880 min, when the equilibrium was achieved. It is worth noting that scaffolds were stable and maintained integrity during the immersion period analyzed in this study.

The release of THC from collagen scaffolds was assessed to determine the delivery trend. As can be observed in Figure 9b, all samples showed a sustained drug release, in which 82%, 45%, and 33% of THC was released from THC2, THC4, and THC6, respectively. It is worth noting that no initial burst of drug release was observed, probably due to collagen–THC interactions by hydrogen bonding (Figure 4), as suggested by FTIR analysis, and due to the compact structure observed by SEM. In order to provide a more sustained release, THC6 scaffold could be preferred.

For a better understanding of the release mechanism, experimental THC release data were fitted to the Korsmeyer–Peppas model and the estimated kinetic parameters are summarized in Table 2. As can be seen, the release data were well-fitted to the Korsmeyer–Peppas model, with R^2^ values higher than 0.9900. The value of *n* indicates the mechanism that describes how the active compound is released from the matrix. In this study, *n* values were higher than 0.89, indicating that the case II release mechanism controls the THC release [56]. These results are particularly interesting since case II release is purely controlled by the swelling of the hydrophilic glassy polymeric system through the relaxation of collagen chains, indicating that the drug transport mechanism is independent of time [57,58].

### 3.5. Mucoadhesive Properties

**Figure 10:**
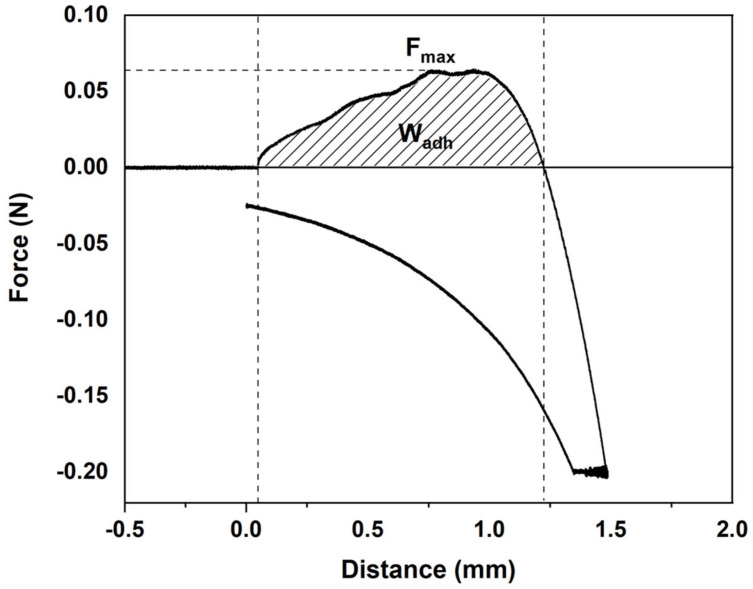
Characteristic load versus deformation curve for THC6 scaffolds.

Considering the potential application of these collagen scaffolds as a local treatment of wounds, a good adhesiveness is essential to immobilize the scaffold, which is the drug-delivery device, on a specific site for targeted release and optimal drug delivery. Mucoadhesion is defined as the adhesion between the polymer and the mucus and intimate contact between them must be formed for the occurrence of mucoadhesion [59]. In this study, mucoadhesive properties of wet collagen scaffolds in contact with type II mucin from porcine stomach were measured. The height of the peak is the maximum force (F_max_) required to separate the probe from the mucin, while the total amount of forces involved in the probe withdrawal from the mucin is the work of adhesion (W_adh_), calculated from the area under the force versus distance curve [60]. All the scaffolds investigated in this study showed positive adhesive forces and, therefore, some form of adhesion occurred between mucus and collagen. Representative load versus deformation curves for THC–collagen scaffolds are displayed in Figure 10. All the recordings from the tensile experiments showed a similar shape.

The good mucoadhesive properties observed on all the samples is the result of the physical interactions among the mucin and collagen, mainly by hydrogen bonding and Van der Waals attraction. These forces are related to the amino acid residues in collagen that form hydrogen bonds with the glycoproteins in mucin [61]. Due to these weak interactions between collagen and mucus, fracture strengths were moderate. The forces produced were mainly due to either mechanical engulfment of the collagen by the mucus or by mechanical penetration of the mucus into collagen crevices. When fracture occurred, collagen almost “popped” out of the mucus layer, as if mucin was not bound to collagen. A failure occurred nearly at the same stage position as the point of initial contact.

As can be seen in Table 3, similar (*p* > 0.05) F_max_ and W_adh_ values were found. The incorporation of THC did not affect F_max_ and W_adh_ values of collagen scaffolds. It is worth noting that different responses can be obtained depending on the analysis type used and the testing conditions employed [62]. Additionally, the mucoadhesive behavior of pure collagen has not been widely studied and, thus, data for comparison have not been found. Therefore, the results of this report should open new avenues of thought regarding the properties of bioadhesive candidates, although further work is needed to promote their application for wound healing.

## 4. Conclusions

Collagen scaffolds with controlled THC delivery and potential for local wound healing were developed by 3D printing. The samples were prepared with high collagen concentration (200 mg/mL) and rheological analysis showed suitable flow behavior to be 3D printed. Neither the addition of THC nor the 3D-printing conditions affected the triple helix structure of native collagen, as observed by SEM and XRD analyses, although physical interactions between collagen and THC were suggested by FTIR analysis. Furthermore, the high water-holding capacity of THC–collagen scaffolds, together with the sustained THC release and mucoadhesive properties, make these scaffolds potential candidates for wound-healing application. In this sense, the sample with 6 wt% THC could be preferred for a more sustained release of the bioactive. In future works, in vitro assays will be carried out to assess the scaffold stability in cell culture conditions as well as cell viability, adhesion, and spreading. Additionally, in vivo tests will be performed to show the therapeutic effect of these scaffolds and their potential in promoting wound healing processes.

## Figures and Tables

**Table 1:** Parameters of Williamson model, master curve shift factors, and shear rate values for collagen doughs.

Sample	η_0_ (Pa·s^−1^)	k (s^−1^)	*n*	R^2^	a_c_	ɣ̇_w_
Control	6764	2.71	0.15	0.996	1	69.05
THC2	4402	1.51	0.42	0.989	0.80	38.43
THC4	2298	1.37	0.31	0.996	0.67	44.47
THC6	2083	1.57	0.28	0.997	0.67	46.94

**Table 2:** Parameters of Korsmeyer–Peppas model for THC release from collagen scaffolds.

Sample	THC2	THC4	THC6
*k*	1.3227	1.4457	0.8651
*n*	1.0660	0.8947	0.9406
R^2^	0.9913	0.9900	0.9944

**Table 3:** Mucoadhesive properties of collagen scaffolds in terms of the detachment force (F_max_) and the work of adhesion (W_adh_). Means followed by the same letter in the same column do not differ statistically among themselves by Tukey test (*p* < 0.05).

Film	F_max_ (N)	W_adh_ (N·mm)
Control	0.0652 ± 0.0035 ^a^	0.0492 ± 0.00304 ^a^
THC2	0.0672 ± 0.0020 ^a^	0.0475 ± 0.00203 ^a^
THC4	0.0681 ± 0.0020 ^a^	0.0494 ± 0.00303 ^a^
THC6	0.0699 ± 0.0030 ^a^	0.0485 ± 0.00233 ^a^

## Data Availability

Not applicable.
